# Triclosan-coated barbed sutures in elective laparoscopic colorectal cancer surgery: a propensity score matched cohort study

**DOI:** 10.1007/s00464-022-09418-0

**Published:** 2022-08-02

**Authors:** Vicente Pla-Martí, José Martín-Arévalo, David Moro-Valdezate, Stephanie García-Botello, Leticia Pérez-Santiago, Ana Izquierdo-Moreno, Ernesto Muñoz-Sornosa, Alejandro Espí-Macías

**Affiliations:** 1 Colorectal Surgery Unit, Department of General and Digestive Surgery, Biomedical Research Institute INCLIVA, Hospital Clínico Universitario, Av. Blasco Ibáñez, 17, 46010 Valencia, Spain; 2 Department of Surgery, University of Valencia, Valencia, Spain

**Keywords:** Surgical site infection, Laparoscopic colorectal surgery, Colorectal cancer, Barbed sutures, Anti-bacterial sutures, Triclosan

## Abstract

**Background:**

Most of the studies published to date which assess the role of antibacterial sutures in surgical site infection (SSI) prevention include heterogeneous groups of patients, and it is therefore difficult to draw conclusions. The objective of the present study was to investigate whether the use of Triclosan-coated barbed sutures (TCBS) was associated with a lower incidence of incisional SSI and lower duration of hospital stay compared to standard sutures, in elective laparoscopic colorectal cancer surgery.

**Method:**

Observational including patients who underwent elective colorectal cancer laparoscopic surgery between January 2015 and December 2020. The patients were divided into two groups according to the suture used for fascial closure of the extraction incision, TCBS vs conventional non-coated sutures (CNCS), and the rate of SSI was analysed. The TCBS cases were matched to CNCS cases by propensity score matching to obtain comparable groups of patients.

**Results:**

488 patients met the inclusion criteria. After adjusting the patients with the propensity score, two new groups of patients were generated: 143 TCBS cases versus 143 CNCS cases. Overall incisional SSI appeared in 16 (5.6%) of the patients with a significant difference between groups depending on the type of suture used, 9.8% in the group of CNCS and 1.4% in the group of TCBS (OR 0.239 (CI 95%: 0.065–0.880)). Hospital stay was significantly shorter in TCBS group than in CNCS, 5 vs 6 days (*p* < 0.001).

**Conclusion:**

TCBS was associated with a lower incidence of incisional SSI compared to standard sutures in a cohort of patients undergoing elective laparoscopic colorectal cancer surgery.

**Graphical abstract:**

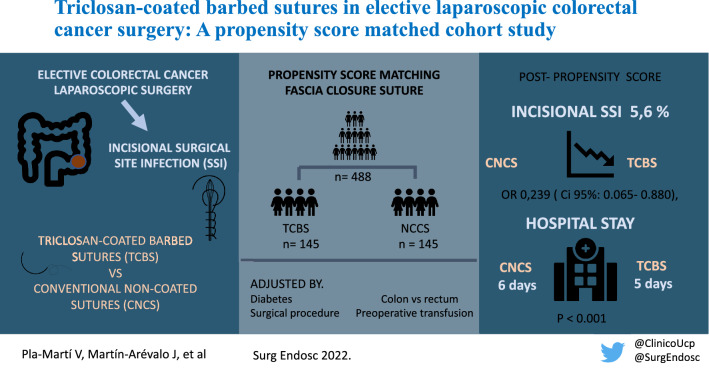

Surgical site infection (SSI) is the most common complication after abdominal surgery. The incidence in elective colorectal surgery can be as high as 20% although there is considerable variation between hospitals [1]. SSI after colorectal surgery increases morbidity and mortality, hospital stay, and healthcare costs [2, 3].

Multiple risk factors related to the patient, surgical procedures, and perioperative care have been described. Some of these are modifiable aspects and constitute important targets with a view to reducing SSI risk [4]. In recent years, the prevention of SSI has become a priority for surgical services and healthcare institutions. In this regard, the introduction of preventive bundle programmes which incorporate various evidence-based preoperative, intraoperative and postoperative interventions has been shown to effectively reduce SSI rates [5–7].

Current evidence demonstrates that the type of suture may determine the risk of SSI [8]. Bacterial colonisation of the suture is one of the risk factors for SSI that has been described [9]. To prevent this colonisation, sutures with antibacterial activity have been developed, and Triclosan is currently the antiseptic most widely used to coat sutures due to the effectiveness demonstrated by in vitro and in vivo studies [10]. Published randomised controlled trials on the role of Triclosan-coated sutures and subsequent meta-analyses have reported conflicting results [11–17]. To date, the largest review of RCT both in terms of number of studies and participants has demonstrated the clinical effectiveness of triclosan-coated sutures in reducing SSI [18]. Most of these studies include different pathologies, surgical approaches, and degrees of contamination, all of which make it difficult to draw conclusions about their effectiveness in elective colorectal surgery.

Barbed sutures provide a homogeneous distribution of tension along the suture line avoiding segmental ischaemia, tissue necrosis and consequently may contribute to decreasing the risk of infection. To our knowledge, there are no studies which evaluate the role of TCBS in reducing SSI in elective laparoscopic colorectal surgery.

The primary objective of the present study was to assess whether the use of Triclosan-coated barbed sutures (TCBS) could reduce the incidence of incisional SSI after elective laparoscopic colorectal cancer surgery. The secondary objective was to determine its impact on hospital stay.

## Materials and methods

An observational retrospective study was carried out in patients who underwent elective colorectal cancer laparoscopic surgery between January 2015 and December 2020. The patients were divided into two groups according to the suture used for fascial closure of the extraction incision, TCBS vs conventional non-coated sutures (CNCS), and the rate of SSI was analysed. This study was approved by the Ethics and Research Committee of our hospital. The inclusion criteria of the study were: age over 18 years, elective surgery, laparoscopic surgical approach, and diagnosis of colon or rectal cancer. The exclusion criteria were: conversion to open approach, abdominal-perineal resection, palliative surgery, postoperative reintervention for reasons other than SSI, postoperative death and loss of follow-up. All patients signed the institution informed consent for laparoscopic colorectal surgery.

Throughout the entire study period, the same bundle of SSI preventive measures was applied [19]. The protocol includes the removal of body hair with clippers 30–60 min before surgery, body hygiene with chlorhexidine soap, antibiotic prophylaxis, antiseptic preparation of the surgical field with 2% chlorhexidine-alcohol, maintenance of body temperature above 36 °C during the procedure, and maintenance of intra- and postoperative blood glucose below 200 mg/dL. The antibiotic prophylaxis (metronidazole 500 mg and cefuroxime 1500 mg or ciprofloxacin 400 mg in case of beta-lactamase allergy) was administered one hour before the intervention, and the antibiotic dosage was repeated for surgeries lasting more than four hours. Mechanical bowel preparation was only used in rectal surgery. Immediately before the abdominal wounds were closed, the surgical instruments and gloves were renewed. A wound protector device was used in all cases.

Surgery was performed following oncological surgical principles. A Hasson trocar was used in the umbilical port and the rest of the trocars were placed according to the type of surgery. The surgical specimen extraction incision was always transverse and located in the right upper quadrant or left iliac fossa according to the location of the tumor. The length of the incision was the minimum necessary required for the extraction of the surgical specimen, and was never greater than 10 cm. Staplers were used for skin closure in all cases. All operations were performed by the same group of dedicated colorectal surgeons, all of whom had more than ten years of experience in laparoscopic colorectal surgery.

TCBS (Ethicon STRATAFIX™ Symmetric PDS™ Plus Knotless Tissue Control Device) has been used after its introduction in our center unless unavailable. Previously, polyglactin 920 CNCS were used for fascial closure in our routine practice.

The definition of SSI was “an infection that occurred within the first 30 days after the intervention that met any of the following criteria: infection of an anatomical plane due to one of the following manifestations: collection, inflammatory signs (pain, swelling, tenderness, redness), dehiscence or positive culture [20]. SSI was classified according to the anatomical plane as: superficial incisional SSI, infection of the skin and subcutaneous tissues; Deep incisional SSI, deep soft tissue infection, (fascia and muscles); and organ / space. [20] In our study only incisional SSI were analysed. Patients were physically assessed in the outpatient clinic by a nurse and the surgeon who operated on the patient. Emergency and clinical records of the patients' primary care physician were also reviewed to avoid missing SII. Follow-up to diagnose SSI was performed within 30 days after surgery.

The study variables were: age, sex, ASA score, comorbidity Charlson’s index, preoperative serum albumin and haemoglobin (Hb), leucocyte and lymphocyte count, body mass index (BMI), diabetes, non-dependent insulin diabetes, insulin-dependent diabetes, location of the primary tumor (colon or rectum), operative time, type of suture used to close the fascia, need for perioperative transfusion and tumoral stage. The outcome variables were the incisional (superficial or deep) SSI of surgical specimen extraction wound and hospital stay. The TCBS cases were matched to CNCS cases by propensity score matching to obtain comparable groups of patients.

### Statistical analysis

Descriptive statistics was performed. Qualitative variables were expressed as total number (relative frequency) and quantitative variables as median (range). The χ^2^ test and Fisher's test were performed when indicated. Odds ratios were calculated. Non-parametric tests (U-Mann–Whitney and Kruskall-Wallis tests) were used. Optimal cut-off points of quantitative variables were calculated based in ROC curves with the highest sensitivity and specificity values.

To obtain two completely comparable group of patients and to reduce possible selection bias, a matched cases study depending on the suture used was performed by matching propensity index adjusted by following variables: diabetes, colon vs rectum location, surgical procedure, and preoperative transfusion. The matching algorithm was the closest neighbor and the estimation algorithm was performed using logistic regression. The matching rate was 1: 1 without restitution. Propensity score matching performed on data from observational studies reduces the risk of including confounding variables. Therefore, many authors point out that the way to objectively extract causality in observational studies would be through the use of these matching techniques [21–25], even with small sample sizes [26].

After matching, a prognostic model of incisional SSI based in binary logistic regression was created with selected patients. Binomial negative regression was calculated to find independent prognostic factors for hospital stay. Multicollinearity was studied with variance inflation index (VIF) in both cases of regression. The *p*-value ≤ 0.05 was considered significant. The analysis was carried out with software R version 3.6.0. Statistical method was supervised by the INCLIVA Research Foundation team. Statistician was not blind to the data. Only the personal data of the patients were anonymised.

## Results

### Outcomes of total cohort before propensity score matching

**Fig. 1:**
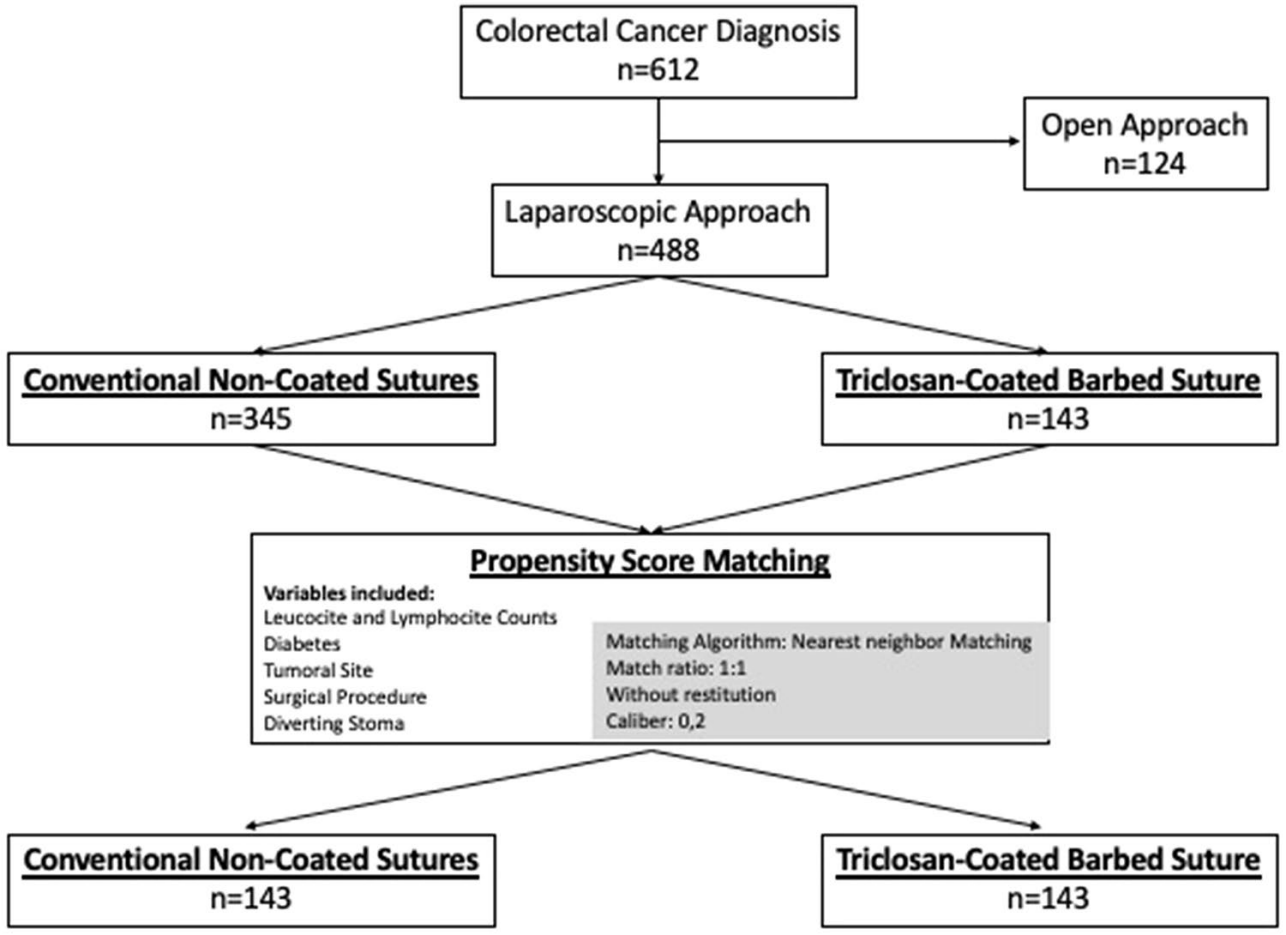
Flow chart of population selection and matching by propensity score

During the study period, a total of 612 colorectal cancer patients underwent laparoscopic elective surgery. Of these, a total of 488 patients met the study inclusion criteria (Fig. 1). The baseline characteristics of the patients included in the study are outlined in Table 1. Overall incisional SSI was observed in 24 (4.9%) patients. Of the quantitative variables studied, the only one that showed statistically significant correlation with incisional SSI was BMI (*p* = 0.026).

**Table 1:** Baseline demographic characteristics of the overall series

Age (years)	70 (21–93)*
Sex (female)	201 (41.2)
BMI	27.24 (16.2–49.9)*
Charlson index	5 (2–13)*
ASA ≥ 3	226 (46.3)
Hb g/dl	12.85 (7.3–17.6)*
Leukocytes (cells/μl)	7.02 (2.1–23.2)*
Neutrophils (cells/μl)	4.24 (1.1–20.1)*
Lymphocytes (cells/μl)	1.8 (0.46–4.39)*
Monocytes (cells/μl)	0.62 (0.22–3.4)*
Diabetes	113 (23.2)
Non-insulin-dependent	89 (18.2)
Insulín-dependent	24 (4.9)
Corticosteroids	2 (0.4)
Smoking	46 (9.4)
Tumor location	
Right colon	170 (34.84)
Transverse colon	19 (3.9)
Splenic flexure	17 (3.5)
Descending colon	26 (5.3)
Sigma	150 (30.8)
Superior rectum	32 (6.6)
Middle rectum	52 (10.7)
Lower rectum	22 (4.5)
Colon cancer	382 (78.3)
Procedure	
Right colectomy	179 (36.7)
Segmental resection	10 (2)
Left colectomy	31 (6.3)
Sigmoidectomy	102 (20.9)
Hartmann procedure	17 (3.5)
Subtotal colectomy	6 (1.2)
Total colectomy	8 (1.6)
Low anterior resection	135 (27.66)
Anastomosis	470 (96.3)
Operative time (minutes)	140 (50–435)*
Preoperative transfusion	30 (6.1)
Postoperative transfusion	37 (7.6)
Neoadyuvant treatment	65 (13.3)
TCBS	143 (29.3)
Incisional SSI	24 (4.9)
Dindo-Clavien	
0	369 (75.6)
I	23 (4.7)
II	85 (17.4)
IIIa	8 (1.6)
VAT	1 (0.2)
IVb	2(0.4)
Diverting stoma	64 (13.1)
Hospital Stay (days)	6 (2–43)*
Tumor stage	
0	37 (7.6)
I	133 (27.3)
II	152 (31.1)
III	141 (28.9)
IV	25 (5.1)

Values in parentheses are percentages, n (%). *BMI* body mass index, *ASA* American Society of Anesthesiologists, *Hb* haemoglobin, *SSI* surgical site infection, *TCBS* Triclosan-coated barbed suture

*Median (range)

The qualitative variables studied that have been shown to increase the risk of incisional SSI are outlined in Table 2.

**Table 2:** Demographic characteristics and outcomes of the TCBS group compared to the CNCS group

	Pre-propensity Score Matching			Post-propensity Score Matching		
	CNCS N = 345	TCBS N = 143	p-value	CNCS N = 143	TCBS N = 143	p-value
Age (years)	70 (21–93)*	70 (46–91)*	0.894	68 (41–92)*	70 (46–91)*	0.336
BMI	27.24 (16.18–49.98)*	27.16 (19.05–37.1)*	0.768	27 (19.8–38.06)*	27.16 (19.05–37.1)*	0.884
Charlson index	5 (2–13)*	5 (2–11)*	0.352	5 (2–13)*	5 (2–11)*	0.797
ASA ≥ 3	157 (45.5)	69 (48.3)	0.580	61 (42.7)	69 (48.3)	0.342
Hb g/dl	12.8 (7.3–17.6)*	12.95 (8.9–17)*	0.596	13.2 (8.8–16.5)*	12.95 (8.9–17)*	0.431
Leukocytes (cells/μl)	7.11 (2.1–15.19)*	6.76 (2.54–23.2)*	***0.031***	6.61 (2.1–15.19)*	6.76 (2.54–23.2)*	0.749
Neutrophils (cells/μl)	4.34 (1.1–11.81)*	4 (1.43–20.1)*	0.124	4.07 (1.1–11.81)*	4 (1.43–20.1)*	0.707
Lymphocytes (cells/μl)	1.83 (0.46–4.39)*	1.72 (0.49–4)*	***0.028***	1.54 (0.52–4.17)*	1.72 (0.49–4)*	0.152
Monocytes (cells/μl)	0.62 (0.22–3.4)*	0.59 (0.24–2.01)*	0.512	0.58 ((0.25–3.4)*	0.59 (0.24–2.01)*	0.303
Diabetes	90 (26.1)	23 (16.1)	***0.017***	28 (19.6)	23 (16.1)	0.440
Non-insulin-dependent	70 (20.3)	19 (13.3)	0.068	25 (17.5)	19 (13.3)	0.325
Insulín-dependent	20 (5.8)	4 (2.8)	0.249	3 (2.1)	4 (2.8)	1
Corticosteroids	2 (0.6)	–	1	1 (0.7)	–	1
Smoking	35 (10.1)	11 (7.7)	0.399	19 (13.3)	11 (7.7)	0.123
Tumor location		21 (14.7%)	0.140			0.248
Right colon	116 (33.6%)			37 (25.87%)	53 (37.06%)	
Transverse colon	14 (4.1%)			8 (5.6%)	5 (3.5%)	
Splenic flexure	13 (3.8%)			4 (2.8%)	4 (2.8%)	
Descending colon	20 (5.8%)			7 (4.9%)	6 (4.2%)	
Sigmoid colon	120 (34.5%)			31 (21.7%)	40 (28%)	
Superior rectum	17 (4.9%)			13 (9.1%)	15 (10.5%)	
Middle rectum	31 (9%)			19 (13.3%)	21 (14.7%)	
Lower rectum	15 (4.3%)			15 (10.5%)	7 (4.9%)	
Colon cancer	282 (81.7%)	100 (69.9%)	***0.004***	96 (67.1%)	100 (69.9%)	0.611
Procedure			** < 0.001**			0.648
Right colectomy	125 (36.3%)	54 (37.8%)		44 (30.8%)	54 (37.8%)	
Segmental resection	6 (1.7%)	4 (2.8%)		1 (0.7%)	4 (2.8%)	
Left colectomy	24 /7%)	7 (4.9%)		9 (6.3%)	7 (4.9%)	
Sigmoidectomy	80 (23.2%)	22 (15.4%)		24 (16.8%)	22 (15.4%)	
Hartmann	13 (3.8%)	4 (2.8%)		4 (2.8%)	4 (2.8%)	
Subtotal colectomy	5 (1.4%)	3 (2.1%)		3 (2.1%)	3 (2.1%)	
Total colectomy	5 (1.4%)	1 (0.7%)		–	1 (0.7%)	
Low anterior resection	87 (25.22)	48 (33.57)		58 (40.6)	48 (33.6)	
Anastomosis	333 (96.5)	137 (95.8)	0.702	139 (97.2)	137 (95.8)	0.749
Operative time (minutes)	135 (50–(435)	146.5 (54–405)	*0.029*	150 (50–435)	146.5 (54–405)	
Preoperative transfusion	28 (8.1)	2 (1.4)	***0.003***	2 (1.4)	2 (1.4)	1
Postoperative transfusion	27 (7.8)	10 (7)	0.752	15 (10.5)	10 (7)	0.295
Neoadyuvant treatment	40 (11.6)	25 (17.5)	0.081	31 (21.7)	25 (17.5)	0.371
Incisional SSI	22 (6.4)	2 (1.4)	***0.020***	14 (9.8)	2(1.4)	***0.003***
Dindo-Clavien			0.513			0.082
0	257 (74.5)	112 (78.3)		95 (66.4)	112 (78.3)	
I	19 (5.5)	4 (2.8)		11 (7.7)	4 (2.8)	
II	61 (17.7)	24 (16.8)		33 (23.1)	24 (16.8)	
IIIa	5 (1.4)	3 (2.1)		2 (1.4)	3 (2.1)	
VIa	1 (0.3)	–		–	–	
VIb	1 (0.3)	–		2 (1.4)	–	
Diverting stoma	36 (10.4)	28 (19.6)	***0.006***	27 (18.9)	28 (19.6)	1
Hospital stay (days)	6 (3–43)	5 (2–24)	** < *0.001***	6 (3–29)	5 (2–24)	** < *0.001***
Tumor stage			0.199			0.311
0	21 (6.1)	16 (11.2)		15 (10.5)	16 (11.2)	
I	94 (27.2)	39 (27.3)		32 (22.4)	39 (22.4)	
II	117 (33.9)	35 (24.5)		51 (35.7)	35 (24.5)	
III	97 (28.1)	44 (30.8)		35 (24.5)	44 (30.8)	
IV	16 (4.6)	9 (6.3)		10 (7)	9 (6.3)	

Pre- and post-Propensity Score Matching; Values in parentheses are percentages, n (%). *BMI* body mass index, *ASA* American Society of Anesthesiologists, *Hb* haemoglobin, *SSI* surgical site infection, *CNCS* Conventional non-coated sutures, *TCBS* Triclosan-coated barbed suture

*Median (range)

Logistic regression based on the variables related to the incisional SSI identified as independent prognostic variables the BMI (*p* = 0.044) and the need for postoperative transfusion (*p* = 0.007).

Overall median hospital stay was 6 days (range: 2–43 days). In the case of patients with incisional SSI, the median stay was 7 days (range: 4–42 days) versus 6 days (range: 2–43 days) (*p* = 0.001) in patients without wound complications. The variables related to hospital stay were age (*p* < 0.001), diabetes (*p* = 0.011), non-insulin-dependent diabetes (*p* = 0.042), chronic renal failure (*p* = 0.038), ASA stage (*p* = 0.010), Charlson´s index (*p* = 0.001), lymphocyte count (*p* < 0.001), lymphocyte neutrophil rate (*p* = 0.001), lymphocyte neutrophil lymphocyte derived rate (*p* < 0.002), pre and postoperative serum Hb value (*p* = 0.016 and *p* = 0.021, respectively), tumor location (*p* = 0.001), operative time (*p* = 0.002), type of suture used (*p* < 0.001), need for postoperative transfusion (*p* < 0.001), and incisional SSI (*p* = 0.001). Predictive variables for hospital stay were age (*p* = 0.0128, VIF = 1.2610), operative time (*p* < 0.001, VIF = 1.05234), type of suture (*p* = 0.0033, VIF = 1.0267), need for postoperative transfusion (*p* < 0.001, VIF = 1.0497), and presence of SSI (*p* < 0.001, VIF = 1.0344).

The study of the characteristics of the groups formed by the two types of sutures showed significant differences in the median operative time (*p* = 0.029), preoperative leukocyte (*p* = 0.031) and lymphocyte count (*p* = 0.028), diabetes (*p* = 0.018), need for pre-transfusion (*p* = 0.003) and colon location of the neoplasm (*p* = 0.005).

The above variables were considered as possible confounding variables and were introduced in the propensity score matching. As a result, the groups obtained after matching were completely comparable.

### Outcomes after propensity score matching

After adjusting the patients with the propensity score, two new groups of patients were generated: 143 TCBS cases versus 143 CNCS cases. Overall incisional SSI appeared in 16 (5.6%) of the patients with a significant difference between groups according to the type of suture used, 9.8% in the group of CNCS and 1.4% in the group of TCBS (OR 0.239 (CI 95%: 0.065–0.880)).

The factors related to incisional SSI were the Charlson´s index (*p* = 0.001), use of CNCS (*p* = 0.003), tumor stage (*p* = 0.020), diagnosis of diabetes (*p* = 0.012) and non- insulin-dependent diabetes (*p* = 0.005). The risk of incisional SSI was higher in patients with a Charlson´s index ≥ 6 (OR: 2.679, 95% CI: 2.679–4.146), as well as in patients with diabetes (OR: 2.685; 95% CI: 1.447–4.980), especially those treated with oral antidiabetic drugs (OR: 3.193; 95% CI: 1.698–6.001) and if CNCS were used (OR: 1.831; 95% CI: 1.465–2.290). The TCBS showed a protective effect on incisional SSI (OR: 0.239; 95% CI: 0.065–0.880) decreasing in 76.1% the risk of incisional SSI. The ability to avoid an incisional SSI was 4.184 times higher with this suture than with CNCS.

**Fig. 2:**
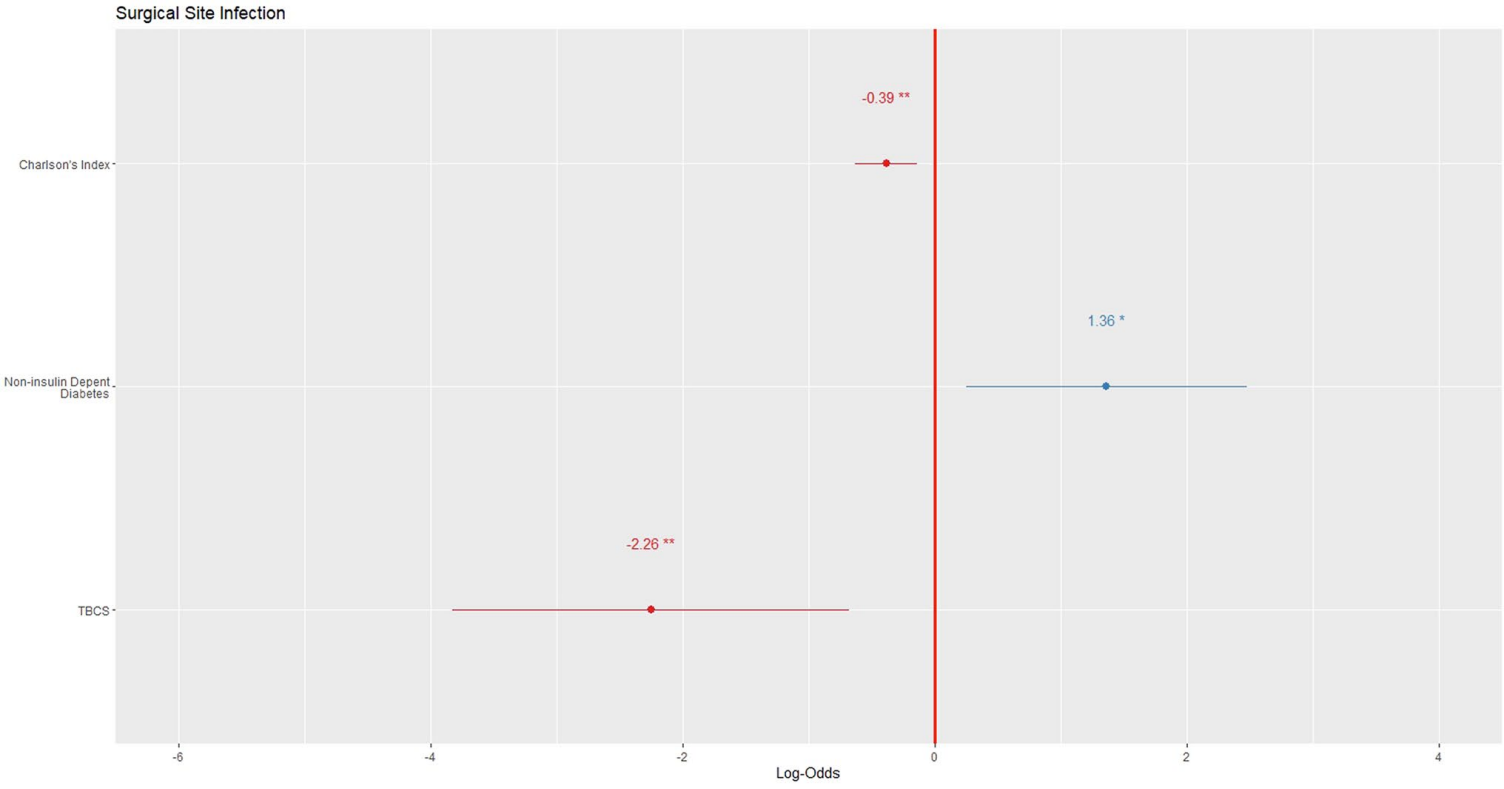
Forest plot of the SSI prediction model after propensity score matching

**Fig. 3:**
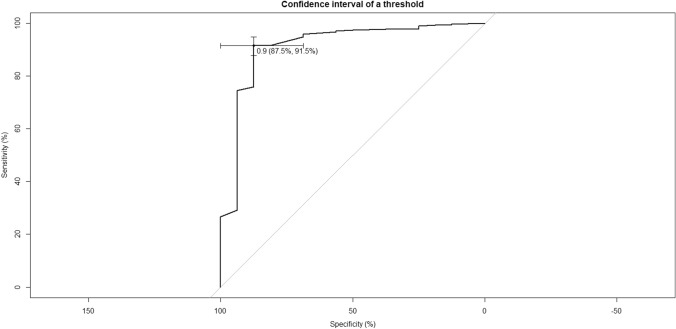
Receiver operator curve demonstrating the ability of the model to predict incisional SSI

In the multivariate analysis, the independent prognostic variables identified were the Charlson’s index (*p* = 0.018), non-insulin-dependent diabetes (*p* = 0.003) and the use of TCBS (*p* = 0.003) Fig. 2. The ROC curve for this model was 91% AUC (95% CI: 0.80–0.98). The sensitivity of the model was 91.5% and the specificity was 87.5%. (Fig. 3).

After matching the cases, the predictive variables for hospital stay were operative time (*p* < 0.001, VIF = 1.0349), type of suture (*p* = 0.0052, VIF = 1.0411) and need for postoperative transfusion (*p* < 0.001, VIF = 1.0523).

## Discussion

The present study demonstrated that the use of TCBS for fascial closure after elective laparoscopic surgery in patients with colorectal cancer decreased the risk of incisional SSI. Overall SSI was 5.6%, with a significant difference between groups depending on the type of suture used, 9.8% in the group of a CNCS and 1.4% in the group of TCBS (OR 0.239 (CI 95%: 0.065–0.880)). Due to the use of TCBS, the risk of SSI decreased by 76.1%. In other words, the probability of avoiding an SSI are 4.18 times more likely with these sutures than with CNCS. On the other hand, the hospital stay was significantly shorter in the TCBS group, which, although not the target of this study, could lead to savings in hospital costs. Since the introduction of TCBS in our hospital, this suture has gradually replaced CNCS in our clinical practice. We have maintained a prospective database for years and have chosen the study period because we have used the same bundle of SSI preventive measures and the same perioperative protocols throughout the study period. The only change was the introduction of TCBS for fascial closure. The TCBS cases were matched to CNCS cases by propensity score matching to obtain comparable groups.

Patients undergoing colorectal surgery have a higher rate of SSI than other abdominal surgical procedures [27]. The laparoscopic approach in colorectal surgery has been shown to be a protective factor for SSI, decreasing the risk compared to laparotomy [4, 28–31]. Several risk factors for SSI in patients with colorectal cancer have been described, relative to both the patient as well as to the surgical procedure or perioperative care. A recent meta-analysis showed that obesity, male gender, diabetes mellitus, ASA score ≥ 3, stoma creation, intraoperative complications, perioperative blood transfusion and operation time ≥ 180 min were significant risk factors for SSI [4]. In our study the factors associated with an increased risk of SSI were BMI ≥ 28, Charlson Index ≥ 6, non-insulin-dependent diabetes mellitus, post-operative transfusions, colon surgery and CNCS use. To avoid bias and to obtain two comparable groups according to whether TCBS or CNCS were used, the aforementioned risk factors were used as covariates in the propensity score matched study. The independent prognostic factors identified were Charlson Index ≥ 6, non-insulin-dependent diabetes mellitus and TCBS. The prognostic model obtained with these factors showed an AUC of 91%. The model created could be useful to identify high-risk patients who might develop SSI.

The outcomes of triclosan-coated sutures in abdominal surgery are controversial. Some studies indicated that triclosan sutures have a protective effect against SSI [13–18] while others showed no beneficial effect [11, 12]. Most of these studies included different pathologies, surgical approaches and degrees of contamination, all of which make it difficult to draw conclusions regarding their effectiveness in elective laparoscopic colorectal surgery. Sandini et al., in a meta-analysis including six RCTs involving 2168 patients undergoing elective colorectal surgery, did not demonstrate a significant SSI protective effect of triclosan-coated sutures over CNCS. The overall SSI rate was 11.7% in the triclosan group and 13.4% in the control group (OR 0.81, 95% CI 0.58–1.13) [32]. The range of SSI incidence was wide (6.8%–16.8%) and the studies included different suture types, wall closure techniques and definitions of SSI, all of which limited the interpretation of the results. Four of the RCTs included exclusively patients with colorectal surgery, open and laparoscopic approach, with contradictory results. Two of them found that triclosan-coated sutures reduced the incidence of SSI [33, 34], while the other two observed no difference with non-coated sutures [35, 36]. Although the level of evidence is moderate to low, several organisations and clinical guidelines recommend the use of triclosan-coated sutures regardless of the type of surgery [37–39].

Barbed sutures allow a homogeneous distribution of tension along the suture preventing segmental ischaemia, tissue necrosis and secondary infection. Furthermore, they avoid the need for knots, reduce incision closure time and provide better waterproofing compared to CNCS. The synergistic effect of the combination of barbed suture and triclosan coating in reducing the incidence of SSI and other complications such as wound dehiscence has been poorly studied to date. A Spanish randomised clinical trial assessed the effect of using triclosan-coated barbed suture for fascial closure on SSI and evisceration after emergency surgery. Incisional SSI was significantly lower (6.4%) in the triclosan-coated barbed suture group than in the triclosan-coated CNCS group (8.9%) and in the uncoated suture group (23.4%, *p* = 0.03). The evisceration rate was 0% in the barbed suture group and 8.9% and 12.8%, respectively in the other groups (*p* = 0.05). The authors concluded that triclosan-coated sutures reduced the risk of SSI and barbed sutures reduced the risk of evisceration and therefore triclosan-coated barbed sutures should be recommended for fascial closure in emergency open surgery [40]. More recently, Johnson et al. have published a multi-institutional, single-arm, retrospective cohort study of patients undergoing open colorectal surgery in which wound closure was performed with triclosan-coated barbed suture. Data were obtained from the Premier Healthcare Database, a database of US hospitals. The overall incidence of wound complications was 7.1%, with the majority being SSIs (6.0%) [41]. [34] To our knowledge, the present study is the first one to have analysed the impact of triclosan-coated barbed suture for fascial closure on the reduction of incisional SSI in patients with colorectal cancer who have undergone elective laparoscopic surgery.

Finally, we acknowledge the limitations of this study. It was an observational study conducted in a single centre and with a selected group of pathologies. In this light its conclusions should be viewed with caution. The retrospective nature of the study could include a possible bias in the selection of the suture for fascial closure. The inclusion only of patients with colorectal cancer could mean that the results cannot be extrapolated to other colorectal pathologies. In the control group, several types of uncoated sutures were used according to the surgeon's preference. In the control group we used polyglactin 920, which is not standard practice for many surgeons and may limit the external validity of the study. In addition, oral antibiotic prophylaxis and mechanical bowel preparation was not used in colon cases and this could reduce the SSI rate. The strengths of this study were that a homogeneous group of patients was included, electively operated on by the same group of colorectal surgeons, with strict protocols for both SSI prevention and perioperative care which remained stable throughout the study period and with extensive patient follow-up. In addition, to reduce potential patient selection bias, propensity score matching was used. This study also provided a prediction model for the risk of wound infection with an area under the curve of 91%, a sensitivity of 91.5% and a specificity of 87.5%.

In conclusion, the use of TCBS in elective laparoscopic colorectal cancer surgery could reduce the incidence of incisional SSI and the length of hospital stays. As this is the most frequently performed procedure in colorectal surgery units, our results could have an important beneficial impact for patients and for the health care system. Well-designed randomised clinical trials are needed to obtain further scientific evidence on the effectiveness of TCBS in reducing SSI and other wound complications in colorectal surgery.
